# COVID-19 Vaccination Attitudes, Perceptions, and Side Effect Experiences in Malaysia: Do Age, Gender, and Vaccine Type Matter?

**DOI:** 10.3390/vaccines9101156

**Published:** 2021-10-09

**Authors:** Mohamed Hassan Elnaem, Nor Hidayah Mohd Taufek, Norny Syafinaz Ab Rahman, Nor Ilyani Mohd Nazar, Che Suraya Zin, Wesley Nuffer, Christopher John Turner

**Affiliations:** 1 Department of Pharmacy Practice, Faculty of Pharmacy, International Islamic University Malaysia, Kuantan 25200, Pahang, Malaysia; hidayahtaufek@iium.edu.my (N.H.M.T.); norny@iium.edu.my (N.S.A.R.); norilyani@iium.edu.my (N.I.M.N.); chesuraya@iium.edu.my (C.S.Z.); 2 Department of Clinical Pharmacy, Skaggs School of Pharmacy and Pharmaceutical Sciences, University of Colorado Anschutz Medical Campus, Aurora, CO 80045, USA; WESLEY.NUFFER@cuanschutz.edu; 3 Retired but Formerly with Skaggs School of Pharmacy and Pharmaceutical Sciences, University of Colorado Anschutz Medical Campus, Aurora, CO 80045, USA; Christopher.Turner@ucdenver.edu

**Keywords:** COVID-19, vaccine, Malaysia, attitudes, perceptions, experience, side effects

## Abstract

This study aimed to investigate the attitudes, perceptions, and experiences of side effects with the COVID-19 vaccines in Malaysia among participants in the National Vaccination Program. A cross-sectional survey was conducted among a sample of vaccine-eligible and vaccinated individuals in Malaysia between May and July 2021. A total of 428 respondents completed the survey. A vast majority (98.6%) of the respondents had registered to be vaccinated. Twenty participants (4.7%) expressed concerns about either registering or receiving the COVID-19 vaccination, mainly due to their uncertainty of vaccine safety. Approximately 77.5% received their vaccinations. Of them, 76.8% had experienced vaccine-related side effects. About 40% of the side effects occurred more with the second dose, particularly those who received the Pfizer-BioNTech vaccine (*p* < 0.001). Pain at the injection site (61.1%) and tiredness (48.8%) were the most reported side effects. Compared to those aged ≥60 years, all age groups were more likely to exhibit vaccine-related side effects; meanwhile, males (OR: 0.51, 95% CI: 0.27–0.93) were less likely to experience side effects than females. Those who received the Sinovac vaccine were at lower risk of experiencing side effects (OR: 0.08, 95% CI: 0.03–0.22) and were more likely to report fewer side effects than Pfizer-BioNTech (*p* = 0.012) and Oxford-AstraZeneca groups (*p*= 0.001). The overall attitudes toward the COVID-19 vaccination program were positive. Several differences in the experiences of vaccine-related side effects, in terms of prevalence and numbers, were attributed to age, gender, and received vaccine type.

## 1. Introduction

Global efforts to face coronavirus disease 19 (COVID-19) rely primarily on the preventive measures implied by individuals to decrease the likelihood of infection transmission [1]. Consequently, the role of COVID-19 vaccinations in complementing the individual preventive measures to face the pandemic has become vital, and vaccination coverage is regarded as critical for maintaining efficient public health measures [2]. Since the availability of COVID-19 vaccines, governments worldwide have exerted tremendous efforts to execute effective procurement and vaccination programs for individuals [3]. However, with the initiation of vaccination programs at the country level, several issues related to vaccines’ acceptability, effectiveness, and safety have become the focus of community concerns [4]. Consequently, the expected and estimated acceptability rates of vaccines vary across countries. In addition, a significant difference in the efficiency of vaccination programs in terms of coverage of eligible communities and addressing vaccine hesitancy concerns have also been reported [5]. Vaccine hesitancy has been identified as a major determinant of vaccine uptake in the community [6]. An Italian study by Napolitano et al. reported that more than 50% of the study population expressed their need to receive further vaccine-related information to help in countering their vaccine hesitancy [7]. In addition, communication, media coverage, and the attitude towards prevention have been reported as primary factors that affect vaccine hesitancy [8].

In Malaysia, vaccines have been distributed through the National Vaccination Program to protect Malaysian citizens from vaccine-preventable diseases since the early 1950s. However, there has been debate in the community about vaccines’ permissibility in general, contributing to vaccine hesitancy in some cases [9]. Malaysia has increased its vaccination rate in response to the ongoing COVID-19 pandemic, to the point where 62% of the population has been fully vaccinated as of 01/10/2021 [10]. The national COVID-19 vaccination program is considered critical for future societal protection and economic recovery planning. According to previous international reports, vaccine acceptability and hesitancy are influenced by various cultural, societal, and awareness factors [11,12]. Consequently, health care providers have implemented several vaccination advocacy initiatives to raise community awareness regarding the critical role vaccines play in the fight against the COVID-19 pandemic [13].

The National COVID-19 Vaccination Program details the policies, vaccine procurement strategies, implementation efforts, and monitoring necessary to contain the COVID-19 pandemic at the national level [3]. The program was divided into three main phases initially planned to cover the entire COVID-19 vaccine-eligible community. Phase 1 of the national vaccination program for COVID-19 has been completed and effectively covered frontliners, including public and private healthcare personnel [3]. Phase 2 has started to cover the remainder of healthcare personnel and senior citizens, while phase 3 is dedicated to the remaining eligible residents of the country not covered in the first two phases [3]. With the program’s implementation, it is essential to explore people’s attitudes, perceptions, and experiences of side effects of their received COVID-19 vaccinations. These findings will be necessary to support the program further and identify the need to foster the success rate of this national initiative [14]. However, little has been reported regarding the attitudes and experiences of a sample of COVID-19-vaccinated and vaccine-eligible individuals concurrently with the ongoing national vaccination program. Therefore, this study aimed to investigate the attitudes, perceptions, and experiences of side effects of COVID-19 vaccines in Malaysia.

## 2. Materials and Methods

### 2.1. Study Design

This cross-sectional study was conducted anonymously online and included a sample of adult individuals in Malaysia concurrently with the national COVID-19 vaccination program. The study was conducted between May and July 2021 using a validated, self-administered survey generated in bilingual Malay and English on Google Forms and then disseminated through social media. Participants were invited voluntarily to participate in the study.

### 2.2. Study Population and Sample Size

Individuals in Malaysia aged 18 and above were accepted to participate in the study, provided that they were eligible to participate in the national COVID-19 vaccination program and they could read and answer the survey questions either in Malay or English. Children and pregnant women were excluded from this study. Regarding pregnancy, there was no conclusive local evidence to include them as potential candidates at the time of survey planning. However, according to the latest guidelines by the Ministry of Health Malaysia, they are now included as a group suitable for vaccination [15]. According to the Raosoft online sample size calculator (Raosoft, Seattle, WA, US), assuming a 5% margin of error, a 95% confidence level, and a 50% response distribution, the required sample size for this study was 377.

### 2.3. Ethical Approval

Ethical approval for this study was granted by the IIUM Research Ethics Committee (IREC 2021-200). Participation information sheets and informed consent forms were included in the online survey form. After reading the information sheet, participants’ agreement to proceed further to the survey items indicated their consent to participate in the study. Participants were informed about the strict confidentiality of their data and the use of their data anonymously for scientific research purposes only. Participants were given the option of withdrawing from the study at any time. Participation was entirely voluntary, with no monetary compensation.

### 2.4. Study Instrument

A self-administered 27-item questionnaire was developed and validated before distributing it to eligible potential participants. A panel of six experts provided inputs to validate the questionnaire. A calculated content validity index (CVI) score of at least 0.83 was achieved for all the finally maintained items. Other items with a CVI of <0.83 were omitted from the final questionnaire. The final questionnaire consisted of two main parts. Part 1 (19 items) covered primary participants’ sociodemographic characteristics, attitudes, and perceptions toward COVID-19 vaccines and the national vaccination program. Meanwhile, part 2 (8 items) covered the experiences of vaccinated individuals regarding the type of received vaccine, experienced side effects and severity, the personal behavior after being vaccinated, and the source of information about COVID-19 vaccines. The questionnaire was designed and distributed via a digital platform. Depending on whether they had been vaccinated or not, the participants had the option of responding only to part 1 (general attitudes and perceptions) or part 2 (experiences). Incomplete responses, on the other hand, were not considered in the data analysis.

### 2.5. Statistical Analysis

Descriptive analysis and frequency assessments were applied to the sociodemographic and practice characteristics of the participants. The chi-square test was used to assess the association between nominal variables. In addition, the Kruskal–Wallis test was conducted to compare the mean rank scores between different groups of participants. Binomial logistic regression was used to ascertain the effects of the variables that showed primarily significant associations, such as age, gender, and vaccine type on the likelihood of experiencing vaccine-related side effects. Statistical analysis was performed using SPSS (SPSS Inc., Chicago, IL, USA) version 22. The level of significance for all comparisons was set at a two-sided *p*-value of less than 0.05.

## 3. Results

### 3.1. General Information of Study Participants

A total of 428 respondents completed the survey. Almost three-fourths of the respondents (71.1%) were 18 to 45 years old. More than half of the respondents (66.4%) were female, and most (85%) were non-smokers. About 50% had a degree or diploma and worked in the public sector (46.7%). The majority (79.2%) reported no chronic diseases, and 70.8% of the respondents had no family members diagnosed with COVID-19 infection. Only 2.8% of respondents had been diagnosed with COVID-19 infection before the vaccine availability. Details of the study participants’ information are presented in Table 1.

### 3.2. Vaccination Attitudes and Adherence to Preventive Measures among Study Participants

As shown in Table 2, almost two-thirds (63.3%) of the respondents rated their adherence level to the recommended standard operating procedures (SOP) (e.g., wearing a mask, physical distancing, and regular use of hand sanitizers) as very high. In addition, about 99% of the respondents had registered for the National COVID-19 Vaccination Program, with a majority (81.5%) registering through the MySejahtera application (MySejahtera, Malaysia) [16]. Moreover, more than 80% of all respondents felt highly confident about the effectiveness of COVID-19 vaccines and believed they had received accurate and sufficient information regarding the COVID-19 vaccines (80.1%). Notably, almost all respondents knew that COVID-19 infection could still happen after completing the recommended vaccination dose (97.2%). Furthermore, they knew that the severity of COVID-19 complications of vaccinated patients is lesser than the unvaccinated COVID-19 patients (94.4%).

### 3.3. Vaccine Hesitancy Prevalence and Reasons

**Figure 1:**
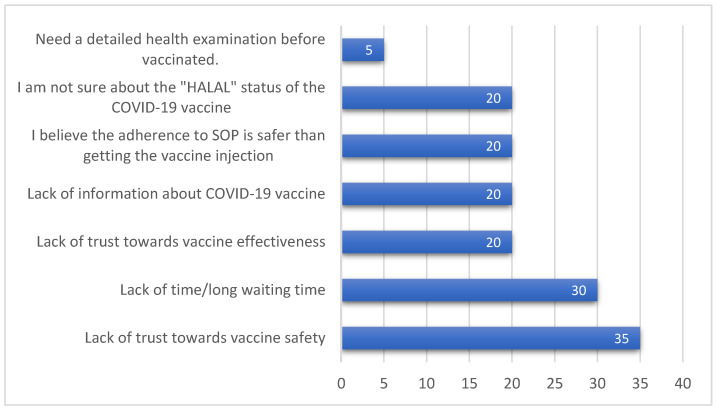
Percentages of common reasons for hesitancy to register or receive the COVID-19 vaccination.

A total of 20 participants (4.7%) expressed their concerns about registering or receiving the COVID-19 vaccination. The uncertainty regarding vaccine safety was the most common reported reason for vaccine hesitancy (*n* = 7, 35%). Figure 1 shows the percentages of reported reasons for vaccine hesitancy among the study participants.

### 3.4. Vaccine Types, Side Effects, and Sources of Vaccine-Related Information

**Figure 2:**
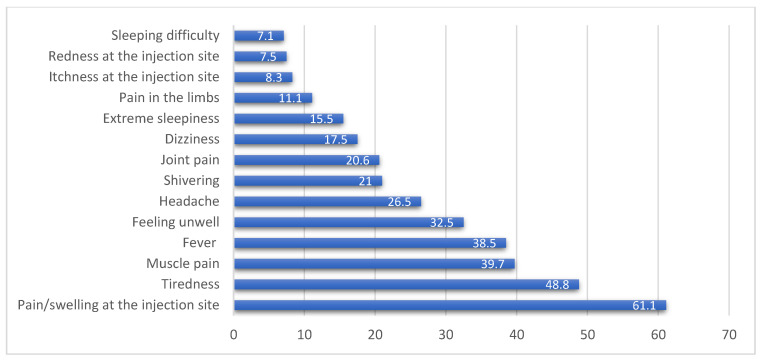
Percentages of commonly reported side effects of the COVID-19 vaccination.

**Figure 3:**
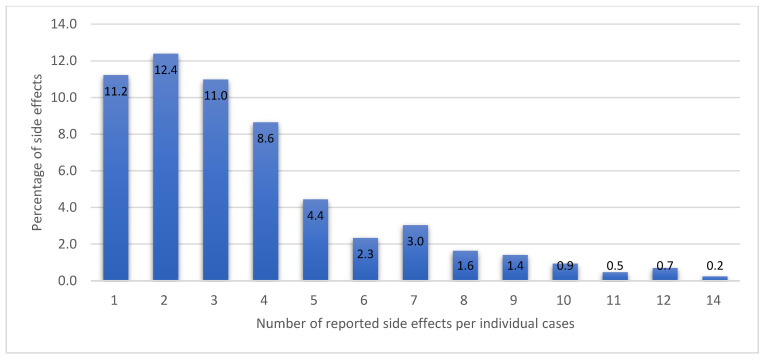
Percentages of the number of reported side effects of the COVID-19 vaccination.

In the present study, among the 428 vaccine-eligible participants, 332 received their vaccination (77.5%). Among the vaccinated respondents, about 50% received the Pfizer-BioNTech (Cominarty^®^) vaccine, followed by 27.1% Sinovac (CoronaVac^®^) and 19.3% Astra Zeneca. A majority (76.8%) of respondents experienced side effects following vaccination. Out of 205 respondents, about 40% reported the side effects occurred more with the second dose. Pain at the injection site (61.1%) and tiredness (48.8%) were commonly reported side effects. Figure 2 shows the percentages of the common side effects reported by the study participants. The distribution of the number of side effects reported after the vaccination expressed in percentages is displayed in Figure 3. Most of the respondents (95%) claimed to feel safer after vaccination, but their adherence to SOP remained the same as before vaccinated. The primary source of information regarding the COVID-19 vaccination was from the Ministry of Health and MySejahtera application (53.7%). Other detailed information about vaccines and sources of information are presented in Table 3.

### 3.5. Gender and Perception on Receiving Adequate Vaccine-Related Information

A chi-square test was used to investigate the possible association between gender and the perception of receiving accurate and sufficient vaccine-related information. The results showed a statistically significant association between gender groups and the perception of receiving accurate and sufficient vaccine-related information (χ2(2) = 6.21, *p* = 0.045).

### 3.6. Types of Vaccines, Side Effects Occurrence with First or Second Doses, Number of Experienced Side Effects

A chi-square test was used to investigate a possible association between the type of received vaccine and the experiencing of vaccine-related side effects after first or second doses. The results showed a statistically significant association between the type of received vaccine and the experience of vaccine-related side effects after first or second doses (χ2(4) = 38.1, *p* < 0.001.) Moreover, a Kruskal–Wallis H test was run to determine if there were differences in the number of experienced side effects between three groups of received vaccines: the “Pfizer-BioNTech (Cominarty^®^)”, “Sinovac (CoronaVac^®^)”, and “Oxford-AstraZeneca (ChAdOx1-S)” groups. Mean rank scores were statistically significantly different between groups (H(2) = 13.662, *p* = 0.001). Pairwise comparisons were performed using Dunn’s (1964) procedure with a Bonferroni correction for multiple comparisons. Adjusted *p*-values are presented. This post-hoc analysis revealed statistically significant differences in the number of experienced side effects between the Sinovac group (Mean rank = 92.76) and the Pfizer-BioNTech group (Mean rank = 127.54) (*p* = 0.012), as well as between the Sinovac group and the Oxford-AstraZeneca group (Mean rank = 144.81) (*p* = 0.001), but not between the Pfizer-BioNTech and Oxford-AstraZeneca groups.

### 3.7. Logistic Regression of Age, Gender, Vaccine Type, and the Experiencing of Vaccine-Related Side Effects

Binomial logistic regression was performed to ascertain the effects of age, gender, and vaccine type on the likelihood that participants experienced vaccine-related side effects. The logistic regression model was statistically significant, (χ2(6) = 64.85, *p* < 0.001). The model explained 27.5% (Nagelkerke R2) of the variance in experiencing side effects and correctly classified 80.3% of cases. Sensitivity was 94.8%, specificity was 31.1%, positive predictive value was 82.4%, and negative predictive value was 63.8%. Only three predictor variables were statistically significant: age, gender, and vaccine type (as shown in Table 4). All age groups, except for the age ≥ 60 years, were at increased likelihood of exhibiting vaccine-related side effects. The younger age group (18–30) had 7.4 times higher odds to experience vaccine-related side effects. Male participants (OR: 0.51, 95% CI: 0.27–0.93) and those who received the Sinovac (CoronaVac^®^) vaccine (OR: 0.08, 95% CI: 0.03–0.22) were at lower risk of experiencing vaccine-related side effects.

## 4. Discussion

The present study reported the attitude and experiences of Malaysian adults following six months of the National Vaccination Program for COVID-19. The findings showed that the general attitudes towards the ongoing vaccination program were positive. There was a high level of adherence to SOP after being vaccinated. The confidence level in the crucial role of vaccines in facing this pandemic was high. However, there were differences in the experiences of the vaccinated individuals regarding prevalence and number of side effects explained by demographic and vaccine type data.

Out of 428 respondents, the majority (71.1%) were from the younger age group below 45. Although the survey was available in two Malay and English languages, most respondents were Malays (92.1%), which is expected as the major race in Malaysia. Furthermore, with the higher number of female respondents (66.4%), more non-smokers in the survey were expected since most smokers in Malaysia were males [17]. However, it was noted that 6.5% of current smokers have also reported their attitude towards vaccines, which is important since they are at higher risk of COVID-19 complications. Furthermore, our findings revealed that approximately 21% of respondents had chronic diseases that may put them at higher risk of COVID-19 infection and complications, implying a need for special care considerations in this patient population [18].

### 4.1. Vaccination Acceptance, Attitudes, and Sources of Information

The majority (86%) of the respondents who attended college or university supported the findings that vaccine acceptance was higher in people with higher education levels and higher income [19]. Digital literacy could be one of the factors to this outcome since it is strongly associated with the utility of information and communications technologies among older adults [20]. However, it is essential to note that the national vaccination program has mainly vaccinated health care practitioners, non-health front liners (e.g., police officers), people with comorbidities, and the elderly population until the completion of the present study. It has been proposed that high-risk populations and disadvantaged groups should be prioritized for vaccination while promoting equity and social justice [21].

Most respondents (98.6%) registered for vaccines through the government application MySejahtera or their employers. The main sources of information about COVID-19 vaccination were the official Ministry of Health website and MySejahtera application (53.7%), followed by social media (22.1%) and others. Nevertheless, we need to acknowledge that there were segments in the Malaysian population manually registering at the vaccination centers or lacking internet access and digital literacy. To achieve herd immunity, greater coverage of technology delivering accurate information will reflect collective attitudes and experiences towards vaccines through communication campaigns by the health authorities [22]. With 32% of respondents having contracted COVID-19 infection or had family members/relatives diagnosed with the disease, these experiences might have influenced our study’s positive attitude towards vaccines. Prior to vaccine availability, a study reported that individuals with COVID-19 infection or their family members did not accept vaccines better than others who had no such experiences [19]. Nevertheless, the influence of personal COVID-19 experience toward vaccines was inconclusive [22].

### 4.2. Vaccination Perceptions and Behaviors in the Post-Vaccination Era

Most of our respondents (97.9%) reported good to very high confidence levels towards vaccine effectiveness. However, about one-fifth did not believe that they received accurate and sufficient information regarding COVID-19 vaccines. An Italian study by Di Giuseppe et al. has highlighted that participants who perceived that they received adequate information about the COVID-19 vaccines were less likely to have concerns about the safety of the vaccines [23]. Transparency in disseminating vaccines and COVID-19 updates by government health officials is vital to prevent distrust towards vaccines. It has been reported that different countries had different COVID-19 vaccines acceptance rates. However, Malaysia was among the highest (94.3%) other than Ecuador (97.0%), Indonesia (93.3%), and China (91.3%), whereas the lowest acceptance rates were found in Kuwait (23.6%), Jordan (28.4%), Italy (53.7), Russia (54.9%), Poland (56.3%), US (56.9%), and France (58.9%) [5]. However, a recent finding from a repeated cross-sectional survey conducted in Hong Kong and China highlighted that the COVID-19 acceptance was lower by 10% in the third wave compared to the initially reported rate in the first wave affected by the growing concerns on vaccine safety and the increasing compliance to personal protective behaviors [24]. Therefore, maintaining acceptance of COVID-19 vaccines has also been linked to the level of trust towards information from government sources and employer’s advice [19], particularly in addressing newly developed vaccines with expedited development or approval with political orientation and interference [22].

Most respondents reported high adherence to standard operating procedures (SOP) to prevent COVID-19 infection. However, a small percentage (8.4%) did not know COVID-19 infection risk following complete vaccination and its severity between vaccinated and unvaccinated individuals. These data indicated that the circulation of the latest information has not effectively reached some communities to control the pandemic effectively. We also found that 1.2% felt safer removing masks after getting vaccinated. In addition, a study reported that people with a positive attitude towards vaccines were more likely to follow strict SOPs than those with negative attitudes [25]. Despite the availability of 13 types of COVID-19 vaccines worldwide with efficacy ranging between 50–95%, preventive behaviors including physical distancing and wearing masks must be continuously enforced by individuals and authorities until effective vaccines are available to overcome the emergence of mutations with coronavirus variants [26]. In addition, health literacy and digital health literacy have been reported independently associated with overall compliance with basic preventive practices [27].

### 4.3. Perceived Concerns towards Vaccination

Less than 5% reported concerns about registering or receiving COVID-19 vaccination (Figure 1). The most common concern was related to the vaccine safety reported previously to hinder vaccine uptake to a certain extent [28]. Some other issues were related to the perceived effectiveness of the vaccine, and willingness to protect others had been reported to influence the acceptance of COVID-19 vaccines [29]. A recent study conducted in Mozambique showed that the concerns about the effectiveness of vaccines were more frequently reported than the safety concerns (52% vs. 29.6%), which underpinned that vaccine hesitancy is significantly affected by the perceived benefit of getting vaccinated [30]. Vaccine hesitancy is a complex global issue involving differences in sociodemographic and external factors, and thus tailored strategies to local intervention must be implemented in the specific population [19,31]. Community intervention has been shown to improve the influenza vaccination rate in specific populations by addressing their concerns [32]. In addition, educational interventions to increase awareness and recommendations of human papillomavirus vaccines has been reported to be effective [33]. In addition, a recent Italian study has examined the willingness to receive COVID-19 vaccines among the university population; the findings demonstrated that males were less likely to have concerns about getting vaccinated, and therefore they were more likely to receive their recommended vaccines [34]. In comparison, our findings showed a statistically significant difference between males and females on their perception of receiving accurate and sufficient vaccine-related information, where males frequently believed that they did not receive adequate vaccine-related information. This point could be of relevant consideration in the vaccine advocacy initiatives.

### 4.4. Experiences of Vaccine-Related Side Effects

Our respondents received vaccines from three different manufacturing companies, which were Pfizer-BioNTech (Cominarty^®^) (53.6%), Sinovac (CoronaVac^®^) (27.1%), and Oxford-AstraZeneca (ChAdOx1-S) (19.3%). Since starting the vaccination program, only 77.5% of respondents have received vaccines, mainly in the last two months (58.5%). The experienced side effects were mainly pain/swelling at the injection site, tiredness, muscle pain, and fever (Figure 2). These were similar to those reported by previous studies [35]. The side effects tend to be more pronounced with the second dose, especially those who received the Pfizer-BioNTech (Cominarty^®^) vaccine. These findings could be further explored in the context of vaccine pre-medications to lessen the severity of side effects. The results showed that males, older individuals (≥60 years), and those receiving the Sinovac (CoronaVac^®^) vaccine were less likely to experience side effects. Previous research conducted among health care workers in Turkey reported that females and younger individuals were more likely to report vaccine-related side effects [36]. Additionally, a previous report during the investigation of mRNA vaccines highlighted those older individuals were less likely to experience vaccine-related systemic side effects [37].

Interestingly, the number of side effects reported with the Sinovac (CoronaVac^®^) vaccine was significantly lower than Pfizer-BioNTech and Oxford-AstraZeneca groups. The Sinovac vaccine is an inactivated vaccine, while Pfizer-BioNTech and Oxford-AstraZeneca are nucleic acid and viral vectored vaccines, respectively. Therefore, the differences in the intensity and pattern of side effects could be attributed to the difference in vaccine type as reported previously in comparing potential COVID-19 vaccine candidates [38]. Notably, preliminary reports on the differences between COVID-19 vaccine candidates indicated that Sinovac vaccines might be associated with approximately five times fewer side effects than the other two tested vaccines [38]. Meanwhile, our findings revealed that Sinovac had a 12 times lower correlation with side effects. The side effects after the second dose were more with the Pfizer-BioNTech vaccine, which correlates with the reported data that the systemic side effects of this type of vaccine tend to increase with the second dose [38]. Although several countries have suspended the Oxford-AstraZeneca (ChAdOx1-S) vaccine, especially among younger people, due to reports of vaccine-induced immune thrombocytopenic thrombosis [39], the vaccine has been used in Malaysia taking the age and the overall vaccination benefit into consideration. The majority of respondents had minor/mild side effects, which were similar to what had been reported by previous studies [40,41], but 10.3% experienced moderately severe to severe side effects, which require further investigation.

In Malaysia, the reported incidence of anaphylaxis following vaccination is quite similar to developed countries. The main allergenic ingredients that have caused anaphylaxis were either polyethylene glycol (PEG) or polysorbate 80. Pfizer-BioNTech and Oxford-AstraZeneca vaccines contained PEG and polysorbate 80, respectively, whereas it is not a case for the Sinovac vaccine. Hence, Sinovac has been used as the alternative vaccine in those who have developed anaphylactic reactions with either Pfizer-BioNTech or Oxford-AstraZeneca vaccines [15]. In addition, the Centers for Disease Control have also reported that severe anaphylaxis after the first dose required immediate treatment and monitoring [41]. Other serious side effects reported in previous studies following the second dose were recurrent Bell’s Palsy from Pfizer-BioNTech [42] and myocarditis with Moderna [43].

The study is not without limitations. The first limitation is related to the generalizability of the results that could not necessarily be expanded to the vaccinated population in Malaysia, considering that our sample, although fulfilling the calculated sample size requirements, was not purposively designed to have national representativeness across the different states in the country. Second, the cross-sectional study design did not provide trends of attitudes and perceptions changes over time. As data were collected online, potential participants with no or poor internet access may be underrepresented. Third, as a survey-based study, it instinctively exposed the risk of recall bias, especially among those who received their vaccines significantly earlier than the time of data collection.

Furthermore, the vaccine hesitancy prevalence should be reported carefully as many study participants have received their vaccination. Furthermore, it is essential to highlight that the reported severity of side effects was primarily based on individuals’ perceptions, not objective measures. Finally, there is also a risk of social desirability bias in this work where participants tend to give more socially desirable answers regarding their vaccination attitudes and perceptions considering the challenging situation of the pandemic in the country at the time of the data collection [44].

## 5. Conclusions

The overall attitudes towards the national vaccination program were positive, with a vast majority registered to be vaccinated. Although vaccine hesitancy was low, the reported concerns must be addressed to ensure a higher success of the vaccination program. There was a high level of adherence to personal protective behaviors after being vaccinated. The confidence level in the crucial role of vaccines in facing this pandemic was high. However, there were differences in the experiences of the vaccinated individuals regarding prevalence and number of side effects explained by demographic and vaccine type data. Younger age had a higher risk of experiencing side effects than the older population (≥60 years). Males had lower odds of developing vaccine-related side effects compared to females. Those who received the Sinovac (CoronaVac^®^) vaccine had lower odds of experiencing side effects and fewer side effects than the other two vaccine types.

## Figures and Tables

**Table 1:** General information of study participants.

	Number	Percentage (%)
**Age**		
18–30	115	26.9
31–45	189	44.2
46–59	68	15.9
60 or more	56	13.0
**Race**		
Malay	394	92.1
Chinese	14	3.2
Indian	3	0.7
Others	17	4.0
**Gender**		
Male	144	33.6
Female	284	66.4
**Smoking status**		
Non-smoker	364	85.0
Ex-smoker	36	8.5
Current smoker	28	6.5
**Highest education level**		
Doctorate	29	6.8
Masters	62	14.5
Degree/Diploma (Uni/college)	224	52.3
Uni Student	53	12.4
Secondary Edu	58	13.6
Informal Edu	2	0.4
**Occupation**		
Public sector	200	46.7
Private sector	89	20.8
Self-employed	38	8.9
Unemployed	46	10.8
Student	30	7.0
Pensioner	25	5.8
**Chronic diseases**		
Yes	89	20.8
No	339	79.2
**Having family members/relatives diagnosed with COVID-19 infection?**		
Yes	125	29.2
No	303	70.8
**Diagnosed with COVID-19 infection before the vaccine availability?**		
Yes	12	2.8
No	416	97.2
Total	428	100.0

**Table 2:** Preventive measures and vaccination attitudes among study participants.

	Number	Percentage (%)
**Please rate your adherence level to the recommended SOP, e.g., wearing a mask, physical distancing, and regular use of hand sanitizers:**		
Fair	1	0.2
Good	15	3.5
High	141	32.9
Very high	271	63.3
**The National COVID-19 Vaccination Program is currently ongoing. Have you registered to be vaccinated?**		
Yes	422	98.6
No	6	1.4
Mode of registration for vaccination *:		
MySejahtera App	349	81.5
Employer	63	14.7
Both (App + Employer)	6	1.4
Others (walk-in/Az voluntary program)	6	1.4
**Please rate your confidence level regarding the effectiveness of COVID-19 vaccines:**		
Low	3	0.7
Fair	6	1.4
Good	53	12.4
High	173	40.4
Very High	193	45.1
**Do you believe that you have received accurate and sufficient information regarding the COVID-19 vaccine?**		
Yes	343	80.1
No	16	3.7
Not sure	69	16.1
**Do you know that COVID-19 infection could still happen after completing the recommended vaccination dose?**		
Yes	416	97.2
No	3	0.7
Not sure	9	2.1
**Do you know the severity of Covid19 complications to vaccinated Covid19 patients is lesser than unvaccinated Covid19 patients?**		
Yes	404	94.4
No	5	1.2
Not sure	19	4.4

* Total responses 424 only.

**Table 3:** Information on vaccine types, side effects, and sources of vaccine-related information.

	Number	Percentage (%)
**Select the type of vaccine that you have received:**		
Pfizer-BioNTech (Cominarty^®^)	178	53.6
Sinovac (CoronaVac^®^)	90	27.1
Oxford-AstraZeneca (ChAdOx1-S)	64	19.3
Total	332	100.0
**The month you received the COVID-19 vaccine:**		
February 2021	3	1.0
March 2021	54	17.6
April 2021	31	10.1
May 2021	39	12.8
June 2021	86	28.1
July 2021	93	30.4
Total	306	100.0
**Did you experience any side effects after receiving the vaccine?**		
Yes	252	76.8
No	76	23.2
Total	328	100.0
**Were the side effects occurred more with the first dose or second dose?**		
More with First dose	50	24.4
More with Second dose	80	39.0
No difference	75	36.6
Total	205	100.0
**Please rate the severity of the side effects that occurred after vaccination:**		
Minor	103	40.9
Mild	69	27.4
Moderate	54	21.4
Moderately severe	24	9.5
Severe	2	0.8
Total	252	100.0
**Choose the most accurate statement that reflects your adherence to SOP “physical distancing and wearing a face mask” compared to before receiving your vaccine:**		
I feel safer removing face mask more frequently compared to before vaccination.	4	1.2
I feel safer, but my adherence to SOP is the same as before vaccination.	305	95.0
I do not feel or practice the SOP related to COVID-19 infection differently.	12	3.8
Total	321	100.0
**What is your main source of information regarding the COVID-19 vaccination?**		
Official website of Ministry of Health and MySejahtera application.	197	53.7
World Health Organization (WHO).	32	8.7
Social media.	81	22.1
Internet sources such as Google & YouTube.	44	12.0
Family and friends	12	3.2
Peer-reviewed scientific papers	1	0.3
Total	367	100.0

**Table 4:** Binomial logistic regression of the impact of age, gender, and the type of received vaccine on the risk of experiencing vaccine-related side effects.

	*p*-Value	Odds Ratio (OR)	95% CI	
			Lower	Upper
Age (18–30)	0.000	7.403	2.640	20.761
Age (31–45)	0.001	3.461	1.617	7.406
Age (46–59)	0.002	4.391	1.720	11.214
Gender (Male)	0.030	0.510	0.278	0.936
Vaccine Type (Pfizer-BioNTech (Cominarty^®^))	0.109	0.447	0.167	1.198
Vaccine Type (Sinovac (CoronaVac^®^))	0.000	0.082	0.030	0.227
Constant	0.002	4.962		
